# Time Does Not Necessarily Make A “Real Expert”

**DOI:** 10.1177/22925503231153441

**Published:** 2023-01-26

**Authors:** Michal Brichacek

**Affiliations:** 1 The First Glance Aesthetic Clinic, Winnipeg, Manitoba, Canada; 2 Department of Surgery, Section of Plastic Surgery, University of Manitoba, Winnipeg, Manitoba, Canada

In a recent editorial on the importance of expertise in plastic surgery, the authors argued that patients seeking aesthetic surgery today have difficulty discerning “real expertise” and specifically targeted surgeons early in practice.^1^ There appears to be a belief amongst some senior plastic surgeons that performing cosmetic surgery is a privilege rather than a right. Some see it as something that must be earned, feeling that you must “put in your time” performing reconstructive surgery for many years before entering the aesthetic field. There is a belief that as a more seasoned surgeon, you are better able to perform cosmetic operations on patients with higher expectations; to many young plastic surgeons, this is antiquated thinking.

Time does not necessarily make a great surgeon and certainly does not make someone a “real expert.” An expert is not just a time-based accolade, but also someone dedicated to their craft who keeps pushing themselves to be better every day.^2^ Depending on their fellowship training, some younger surgeons may potentially be more qualified to perform certain operations compared with some of their more senior colleagues. Surgery requires a certain level of training and experience, but natural ability also plays a role. Surgery is a skill, and some people are better at it than others. Some younger surgeons have seen and corrected post-operative aesthetic problems in patients previously operated on by senior surgeons with years of experience.

Less-than-ideal results may be more common for senior surgeons who spent years doing reconstructive surgery and then decided to change their practice to aesthetics much later in their careers. Twenty years of experience doing mainly hand and breast surgery does not make someone more of an “expert” in doing facelifts. Some of these surgeons will still perform these surgeries using techniques they learned decades ago during their residency training.

Expertise matters. However, there are ways of gaining expertise aside from our own trial and error. It has been wisely said that “you must learn from the mistakes of others—you will never live long enough to make them all yourself.”^3^ Is it possible that a young surgeon who completed a craniofacial or aesthetic surgery fellowship, visited surgeons around the world learning the latest techniques, and then began performing these procedures early in their career developed proficiency at an earlier stage?

In some cases, more senior surgeons may be stuck in their ways and less open to change. They might feel that they have been doing facelifts the same way for years and are getting fair results, so why should they change their techniques? Perhaps some younger surgeons are more open to innovating and trying new techniques. The field of aesthetic surgery is constantly changing, and it is important to embrace change when it leads to better results. Some younger surgeons have already embraced new techniques and do things differently than what they learned in residency and fellowship only a few short years ago. They have done this to improve their outcomes, but it does not mean that they are not appreciative and grateful for the training opportunities from their mentors.

Both young and experienced surgeons should not be misrepresenting themselves and claiming things that are simply not true. That is unethical. This applies to surgeons who pay to have lofty articles written about themselves or those who pay to be listed as a top-ranked surgeon by some magazine or website, as has been duly noted.^1^

Self-promotion is nothing new, and ultimately, results do speak for themselves. All plastic surgeons can post photos demonstrating their work and outcomes. Are objective results not an accurate measure of a surgeon's skill and expertise? If some senior surgeons do not want to market themselves outside of “word of mouth,” that is their choice. Younger surgeons may be more open to embracing new ways of promoting themselves. If a younger surgeon can show that they are achieving good results and have happy patients, then older surgeons complaining about them just makes it seem that they are bitter that they did not “earn their place.”

Rather than complaining amongst ourselves, we, as plastic surgeons, should be more concerned about other specialties making inroads into the field of aesthetic surgery.^4^ If we do not support our younger surgeons in performing aesthetic surgery, we will continue to lose this to other specialties. Why do we lack adequate aesthetic training options in Canada? Why do some of our most well-known experts in Canada not have fellowship programs? Why do young plastic surgeons find it difficult to get a job if they have aesthetic training? These are all things we need to seriously consider if we want plastic surgeons to continue to be leaders in aesthetic surgery in the future.
